# Response Surface Methodology: Photocatalytic Degradation Kinetics of Basic Blue 41 Dye Using Activated Carbon with TiO_2_

**DOI:** 10.3390/molecules26041068

**Published:** 2021-02-18

**Authors:** Emmanuel Kweinor Tetteh, Elorm Obotey Ezugbe, Dennis Asante-Sackey, Edward Kwaku Armah, Sudesh Rathilal

**Affiliations:** Green Engineering and Sustainability Research Group, Department of Chemical Engineering, Faculty of Engineering and The Built Environment, Durban University of Technology, Durban 4001, South Africa; elormezugbe.ee6@gmail.com (E.O.E.); ingsackey@gmail.com (D.A.-S.); edwardkarmah@gmail.com (E.K.A.); rathilals@dut.ac.za (S.R.)

**Keywords:** activated carbon, box-behnken design, dye, response surface methodology, TiO_2_ photocatalyst

## Abstract

Water decontamination still remains a major challenge to some developing countries not having centralized wastewater systems. Therefore, this study presents the optimization of photocatalytic degradation of Basic Blue 41 dye in an aqueous medium by an activated carbon (AC)-TiO_2_ photocatalyst under UV irradiation. The mesoporous AC-TiO_2_ synthesized by a sonication method was characterized by X-ray diffraction (XRD) and Fourier-transform infrared (FTIR) spectroscopy for crystal phase identification and molecular bond structures, respectively. The efficiency of the AC-TiO_2_ was evaluated as a function of three input variables viz. catalyst load (2–4 g), reaction time (15–45 min) and pH (6–9) by using Box-Behnken design (BBD) adapted from response surface methodology. Using color and turbidity removal as responses, a 17 run experiment matrix was generated by the BBD to investigate the interaction effects of the three aforementioned input factors. From the results, a reduced quadratic model was generated, which showed good predictability of results agreeable to the experimental data. The analysis of variance (ANOVA), signposted the selected models for color and turbidity, was highly significant (*p* < 0.05) with coefficients of determination (*R*^2^) values of 0.972 and 0.988, respectively. The catalyst load was found as the most significant factor with a high antagonistic impact on the process, whereas the interactive effect of reaction time and pH affected the process positively. At optimal conditions of catalyst load (2.6 g), reaction time (45 min), and pH (6); the desirability of 96% was obtained by a numerical optimization approach representing turbidity removal of 93% and color of 96%.

## 1. Introduction

With the recent industrial revolution, large volumes of wastewater produced by textile industries containing high levels of pollutants such as dyes and pigments, pose a great threat to human health and the environment. Globally, it has been estimated that about 800,000 tons of dyes and pigments are produced per year, whereby about 25% of them end up in the environment via wastewater discharges [1,2]. This poses a great threat to the environment and a serious health concern. Thus, in the textile industries, during the dyeing processes, large volumes of water are used with the end-product wastewater containing high levels of organics and toxic compounds. These wastewaters, when discharged into water bodies without treatment, end up affecting the dissolved oxygen levels for aquatic life [3].

Subsequently, dyes at concentrations even as low as 1 ppm can contribute to high strength color associated with reduced oxygen dissolution and sunlight diffusion which is necessary for aquatic life [3,4,5]. Therefore, there is the need to treat these colored effluents to remove the dyes before discharging them into water bodies [5]. Basic Blue 41 dye (BB41) is a dye typically used for dyeing wool and silk [4,6]. Discharges of effluents with the dye has recently been reported as a public health concern causing eye burns, nausea, vomiting, and diarrhea [2,7].

Finding an effective treatment method for wastewater-containing dyes is paramount as numerous treatment technologies have been established for the discoloration and degradation of dyes in wastewater [8,9,10]. Some of these techniques include adsorption, reverse osmosis, ultrafiltration, chlorination [9,10] and biological methods, such as aerobic and anaerobic treatments [11]. Nonetheless, most of these traditional techniques eliminate pollutants by simply transferring them from one phase to another without any conversion into environmentally-friendly end products [12,13]. These created intermediates usually require some form of secondary treatment or polishing step. Advanced oxidation processes (AOPs) such as Fenton, photo-Fenton catalytic reactions, UV/H_2_O_2_, and UV/O_3_ are effective methods for treating wastewater-containing dyes [14,15]. Comparatively, the removal of organic contaminants via photocatalytic degradation has proven to be more efficient than these conventional processes as it results in the total mineralization of a wide range of organic pollutants and the fact that it produces harmless compounds such as CO_2_ and H_2_O [13,15].

Among the numerous semiconductors (TiO_2_, ZnO, WO_3_, etc.), titanium dioxide (TiO_2_) is a promising photocatalyst due to its unique biological and chemical stability, low price and ready availability [9,16,17,18]. Narayanasamy and Murugesan [19] and Natarajan, et al. [20], reported photocatalytic degradation of yellow dye using TiO_2_ thin films under visible light, where the degradation efficiency decreased with an increase in catalyst load. Similarly, other studies have exposed TiO_2_ photocatalysts to UV light for discoloration of different types of dyes in wastewater such as Direct Red 23, Toluidine Blue, Safranin Orange, and Methylene Blue [21,22,23]. Apart from the catalyst load, several other factors which includes pH, temperature, wavelength, reaction time, and the pollutant concentration can influence the photocatalytic efficiency [24,25,26]. In response to this fact, optimization becomes an option to improve photocatalytic degradation efficiency. Herein response surface methodology (RSM) is proven as an effectual tool for process optimization with multi-complex operational factors [26,27,28]. A Box Behnken design (BBD), a type of RSM, has shown to be very efficient as compared to the central composite design (CCD) in estimating the correlation coefficient (*R*^2^) for a specified model, reduced experimental errors and good data fitness in the design, modeling and optimization of a process [24,28].

However, after degradation it is difficult to settle immobilized TiO_2_ nanoparticles in solution, so enhancing water clarity requires an extended period of time [21,23]. The probability of this resulting in the formation of non-degradable toxic intermediates seems very high, which is, therefore, increasing environmental and human health concern [18,22]. Ideally, due to the adsorption potential of activated carbon (AC), combining AC and photocatalytic systems [19] is an option for treating pollutants and minimizes the cumulative cost of disposal of wastewater residue. To the best of our knowledge, there is little knowledge in the literature on the use of activated carbon (AC) doped with TiO_2_ for photocatalytic degradation of BB41 via RSM optimization techniques. Therefore, this study presents the optimizing of photocatalytic degradation of BB41 in an aqueous medium by AC-TiO_2_ photocatalyst under UV light irradiation using RSM. Furthermore, the kinetic degradability of raw TiO_2_ and AC-TiO_2_ is comparatively explored.

## 2. Materials and Methods

### 2.1. Regeants and Materials

#### 2.1.1. Chemicals

Basic Blue 41 (BB41, CAS Number 12270-13-2) of 40% dye with absorbance (λ = 617 nm) and molecular weight (C_20_H_26_N_4_O_6_S_2_) of 482.57 g/mol, hydrochloric acid (HCl) and sodium hydroxide (NaOH) were purchased from Sigma Aldrich (Durban, South Africa). All chemicals used, unless otherwise stated, were of analytical grade. The locally manufactured TiO_2_ supplied by Huntsman Tioxide (Durban, South Africa), is made up of anatase (75%) and rutile (25%) with mean particle size of 25 nm, specific surface area and density of 48 m^2^/g and 4.26 g/cm^3^, respectively. Activated carbon (AC) with specific surface area, pore-volume, and an ash content of 1576 m^2^/g, 0.967, and 3%, respectively, were supplied by a local South African industry (RTB Chemicals, Pietermaritzburg, South Africa).

#### 2.1.2. Synthetized Textile Effluent

A stock solution of 2.5 M BB41 was prepared by diluting 30.16 g of the dye in 5 L deionized water coupled with 20 L of local wastewater [3]. HCl and NaOH were used for pH adjustment, measured with a Hannah pH—meter HI98130 (Hanna Instruments, Woonsocket, RI, USA). The main composition of the synthesized effluent was pH (7.55), COD (176.87 mg/L), color (54.8 Pt.Co) and turbidity (12.28 NTU). The COD and color was measured with a HACH DR 3900 (Hach Company, Loveland, CO, USA), while turbidity was measured by using a HI 98703 Portable Turbidimeter (Hanna Instruments, Veneto, Italy).

#### 2.1.3. AC-TiO_2_ Composite Preparation and Characterization

The AC-TiO_2_ nanocomposite was prepared by the co-precipitation method assisted with a low temperature of 90 °C [29]. In brief, 30 g and 10 g of AC and TiO_2_, respectively was added to 200 mL of deionized water and sonicated at 45 kHz for 1 h. Successive overnight drying at 100 °C resulting in the AC-TiO_2_ composite was washed with deionized water/ethanol, filtered, and dried for 1 h at 110 °C to remove any excess impurities. Preceding, the AC-TiO_2_ was calcined at 600 °C for 1 h at a ramp speed of 15 s. The mineralogical constituent and phase magnitude of the composite was determined by X-ray diffraction (XRD) equipment (D8 Advance AXS, Bruker, Elmford, NY, USA) coupled with J-J scan and copper anode filter (Cu-Kα radiation: λ = 1.5406 Å) at the rate of 0.033°/min over a range of 5 to 70° (2θ). Fourier Transform Infrared spectroscopy (FTIR) analysis was carried out to identify the functional groups of the AC-TiO_2_ composite. This was carried out with a FTIR 8400 FTIR-spectrometer (Shimadzu, Columbia, MD, USA) in the wave-number range of 300–4000 cm^−1^ with a resolution of 4 cm^−1^.

### 2.2. Experimental Procedure

#### 2.2.1. Photocatalytic Degradation

The photocatalytic degradation experiment was carried out in batch-wise mode using six 1 L reactors coupled with UV-T8 blacklight—blue bulb of wavelength 400 nm and power of 18 W, (Philips, Eindhoren, The Netherlands), under constant stirring at a speed of 150 rpm [24]. The reactor was placed in a closed chamber with the UV-light placed at a distance of less than 2 cm. To establish the photocatalytic kinetics, 3 g of each catalyst (TiO_2_ and AC-TiO_2_) was added to 500 mL dye solution. The irradiation time (10–60 min) was varied while the solution was constantly stirred. At each time interval of 10 min, supernatant samples were collected with a syringe and filtered through a Whatman filter (Grade 5; 2.5 µm). The kinetics degradation efficiency was monitored in terms of the COD removal, by using Equation (1). To estimate the rate of reaction constants for the first-order kinetic model (2), a plot of $ln\frac{C_{o}}{C}\;vs\;t$ was developed:

(1) $$\mathrm{Removal}\;\mathrm{efficiency}\;(\%)\;=\;\frac{C_{0}-\;C_{f}}{C_{0}}\;\times \;100$$

(2) $$ln\frac{C_{o}}{C}=\;k_{1}t$$

where, $k_{1}$ is the first-order rate constant (min^−1^),$\;\;C_{0}$ is the initial COD concentration, t is time and $C_{f}$ is the concentration at the specified time.

#### 2.2.2. Design of Experiments

**Figure 1:**
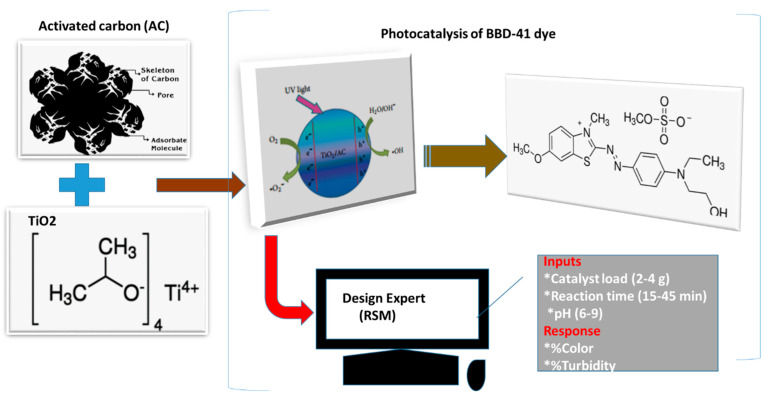
Schematic representation of the photocatalytic process via response surface methodology.

The photocatalytic efficiency (Figure 1) was investigated based on the experimental design with the three operating factors (Table 1). This was carried out by using the Box-Behnken design (BBD) by Design-Expert software (version 11.1.0.1) (Stat-Ease, Inc., MN, USA). The BBD is a factorial combination with a minimum of three factors that were studied under the randomized response surface methodology. The effect of three independent factors such as A: catalyst load (2–4 g), B: reaction time (15–45 min), and C: pH (6–9) was examined on photocatalysis with turbidity (Y_1_) and colour (Y_2_) as the responses. All the experiments were carried out in duplicate and average values obtained are reported.

From Table 1, the total number of the experiments was estimated by Equation (3). Seventeen experimental runs were obtained according to a three-level scale such as low (−1), medium (0), and high (+1) coupled with five center points (Cps). The responses (Y_1_ and Y_2_) were expressed as a function of the input variables (A, B, and C) by fitting the data obtained on a polynomial quadratic model (4). The empirical model developed depicts the influence of the input variables on the response within the design space [24]. Analysis of variance (ANOVA) was employed to evaluate the significance of the results statistically by considering *p* < 0.05 [26]:

(3) $$NE=\;k^{2}\;+\;k\;+\;Cp$$

where  NE, k and Cp  represents the total number of experiments, the number of factors, and the replicated number of center points. To analyze the pivotal relationship between the dependent and independent variables, the polynomial quadratic expressed in Equation (4) was utilized:

(4) $$Y=\;\beta_{0}\;+\;\overset{K}{\underset{i=1}{\sum }}\beta_{i}x_{i}+\overset{K}{\underset{i=1}{\sum }}\beta_{ii}x_{i}^{2}+\overset{K-1}{\underset{i=1}{\sum }}\overset{K}{\underset{j=2}{\sum }}\beta_{ij}x_{i}x_{j}+\varepsilon$$

where, Y is a response variable, $\beta_{0}\;$ is a constant, $\beta_{i}$ is the regression coefficient, *k* is the number of independent variables, and ε is the unknown error constant. Also, the coded values of the independent variables were expressed by Equation (5):

(5) $$x_{i}=\frac{X_{i}-X_{io}}{\Delta X_{i}},\;i=1,2,3,\ldots k$$

where, *x_i_* are the coded values and *X_i_* the real values of independent variables, *X_io_* the real value in the center plane, and $\Delta X_{i}$ the step change. The model’s overall prediction efficiency is commonly assessed by the coefficients of determination (*R*^2^), which can be calculated by Equation (6):

(6) $$R^{2}=\frac{SS_{residual\;error}}{SS_{total}}\;\;$$

This coefficient is used to measure the total response variation of predicted values from the mean response. However, the addition of variables to the model increases *R*^2^ values irrespective of its statistical significance.

## 3. Results and Discussion

In this study, photocatalytic degradation of BB41 dye with a hybridized photocatalyst (AC-TiO_2_) was optimized using response surface methodology. This feasibility study was carried out to strengthen the knowledge of using TiO_2_ for photocatalysis and absorption processes. The results obtained are presented under Section 3.1, Section 3.2 and Section 3.3, respectively, denoting the physiochemical properties of the AC-TiO_2_ composite, photocatalytic kinetics, and response surface methodology.

### 3.1. Physiochemical Properties of the Photocatalyst

**Figure 2:**
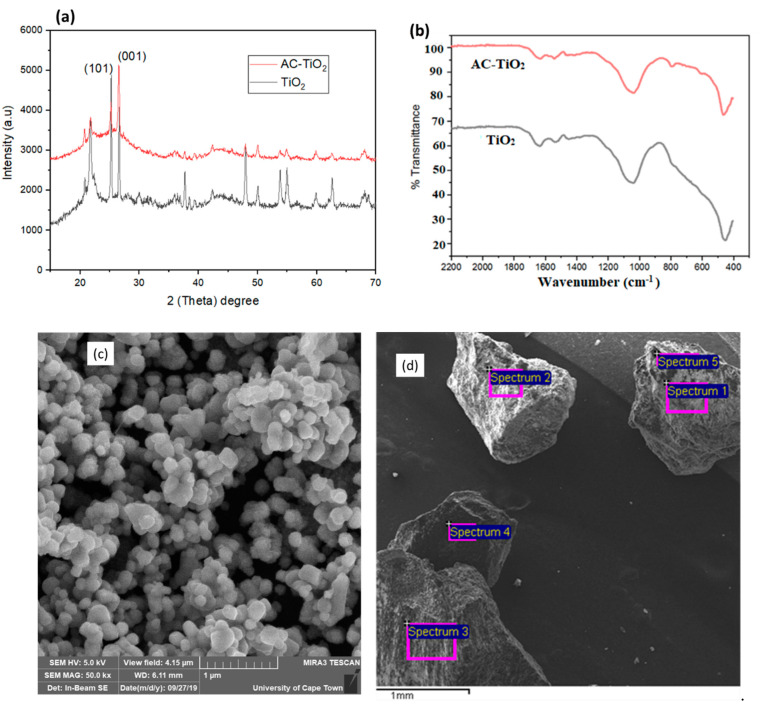

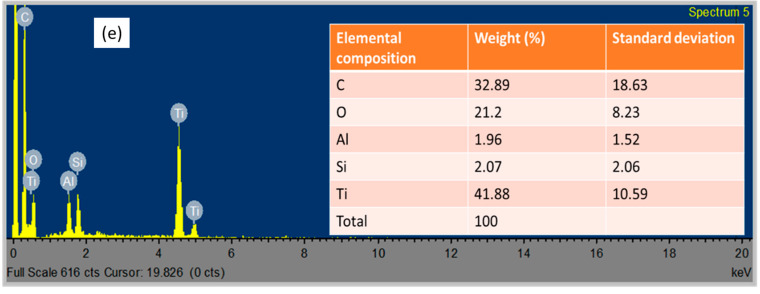
Spectra of (**a**) XRD and (**b**) FTIR analysis for TiO_2_ (black) and AC-TiO_2_ (red); (**c**) SEM and (**d**) EDX image of AC-TiO_2_ and (**e**) EDX spectra and elemental composition of AC-TiO_2._

Figure 2 presents the physiochemical results obtained. The XRD (Figure 2a) and FTIR (Figure 2b) spectra of the TiO_2_ and AC-TiO_2_ composite, confirm the successful impregnating of the TiO_2_ on the AC surface [15,30]. The crystalline phase of the catalysts (TiO_2_ and AC-TiO_2_) as observed by the XRD (Figure 2a) was seen to be very prominent, with anatase corresponding to the JCPDS Files No. 21-1272. At the scanning range of 20° > 2θ > 70°, the peaks observed at various diffractions of 27.47°, 36.07°, 37.80°, and 69.0° assigned to (001), (021), (210) and (220) reflects the rutile phase of TiO_2_. Whereas, the reflections of the peaks at 2θ of 25.4°, 48.02°, 50°, 54.19°, 55° and 62.72° were dispensed to (101), (110), (200), (211) (105) and (103) of the anatase plane. The rutile phase intensity is considerably less than that of the anatase phase [29,30]. The average crystalline size (28 nm to 22 nm) was then determined by Scherrer’s equation (Equation (7)) with the crystalline plane of (101) and (001) for anatase and rutile, respectively:

(7) $$D=\frac{K\delta }{\beta Cos\;\theta }$$

where δ is the wavelength of X-ray radiation (CuKα = 0.15406 nm), k is a constant taken as 0.9. *β* is the line width at half maximum height (FWHM) of the peak (determined using Origin software), and *θ* is the diffraction angle of the most intense peaks of rutile (27.47°) and anatase (25.4°).

Figure 2b presents the FTIR spectra ranged with a wavenumber of 400–2200 cm^−1^. The spectrum also reveals the adsorption bands between the reactive hydroxyl groups and the hydrophobic and hydrophilic nature of TiO_2_ [12,15]. This is associated with the vibration and stretching modes of the TiO_2_ and different oxygenated functional groups. The peaks at 1400–1600 cm^−1^ represent the stretching of hydroxyl (OH) and carboxyl (C=O) groups which might have been caused by either water moisture or the titanium carboxylate [8,30]. There is evidence of a shift in the OH vibration band towards a lower wavenumber (<800 cm^−1^) of the AC-TiO_2_ spectra as compared to the TiO_2_ [15,29]. This might be due to the acid-base interaction of the OH group used in synthesizing the AC-TiO_2_ composite [29]. Lastly, the broad stretching peaks around 400–600 cm^−1^ is also the representation of the TiO_2_ bonding in the composite [8,12]. The surface morphology of the AC-TiO_2_ is represented by the SEM (Figure 2c) and EDX (Figure 2d) images. With scanning microscopy magnification (Figure 2c) of 50 kx and binding energy of 5 kV, grain size of 4.15 μm was viewed under the scale of 1 μm and working distance (WD) of 6.11 mm. EDX image (Figure 2d) under scale of 1mm revealed the TiO_2_ spectra (Figure 2e) at the binding energy of 0.5, 4.5 and 5 keV. This affirmed the success of incorporating the TiO_2_ with the AC. Also the elemental composition of the composite (AC-TiO_2_) was distinctively defined as Ti (41.88%) > C (32.89%) > O (21.2%) > Si (2.07%) > Al (1.96%).

### 3.2. Photocatalytic Degradation Kinetics

**Figure 3:**
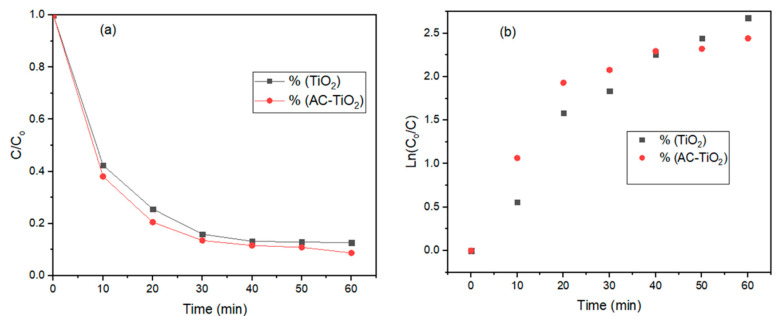
Comparative effect of TiO_2_ (black) and AC-TiO_2_ (red) on (**a**) decomposition of COD and (**b**) First-order kinetic model.

Figure 3 presents the comparative effects of TiO_2_ and AC-TiO_2_ (Figure 3a), as well as their photocatalytic kinetics (Figure 3b) on the removal of COD from the effluent. Chemical oxygen demand (COD) as the organic strength of effluent, in this instant, was used to measure the efficacy of the catalyst. This is also attributed to the amount of oxygen required for the oxidation of the dye into CO_2_ and H_2_O, with the possible reactions presented in (8) and (9) [31,32]. To estimate the reduction values of the COD, the before and after treatment results obtained were used. Figure 3 denotes the mineralization of the dye organics (COD) along with an increase in the reaction time (10–60 min). However, rapid degradation was observed after the 30 min irradiation (Figure 3a), where it became stabilized within 40–50 min with a slight drift towards 60 min. The maximum degradation of 93% and 96% COD removal, respectively, by TiO_2_ and AC-TiO_2_ was obtained at the 50 min irradiation:

(8) $$\begin{array}{c} H_{2}O+h^{+}\overset{\mathrm{AC}-{\mathrm{TiO}}_{2}^{*}}{\to }{\;\mathrm{OH}}^{*}+H^{+}\\ {\mathrm{OH}}^{*}+\mathrm{BB}41\;\mathrm{molecule}⟶\mathrm{intermediates}⥅{\mathrm{CO}}_{2}↑\;+H_{2}O+\mathrm{mineral}\;\mathrm{salts} \end{array}$$

Consequently, activation of TiO_2_ nanoparticles by the UV-light produced reactive species (OH), which facilitated the degradation of organics in the solution [12,33]. As shown in Figure 3b, the degradability followed the first-order kinetics for irradiation time less than 60 min. The kinetic rate constants were estimated as 0.0322 min^−1^ and 0.0366 min^−1^ for TiO_2_ and AC-TiO_2_, respectively, at regression coefficient (*R*^2^) values of 0.889 and 0.9188. The recorded residual sums of squares (SSR) were 0.7704 and 0.691 for TiO_2_ and AC-TiO_2_, respectively. It was found that the degradability of COD was a function of the irradiation time, thus confirming previous kinetic studies on the degradation of organic pollutants in wastewater that generally followed Langmuir-Hinshelwood (L–H) kinetics [12,31].

### 3.3. Response Surface Methodology (RSM)

The experimental runs, as mentioned earlier, were designed by the RSM via the BBD to investigate the key operating parameters such as catalyst load (A), reaction time (B), and pH (C). The BBD matrix obtained with 17 randomized sets of experimental runs, along with the response and predicted results obtained, is presented in Table 2. The maximum removal of the contaminants (colour and turbidity) obtained was within the range of 90–95% for the actual results with a deviation of less than 5% from the predicted values. The experimental data (Table 2) was fitted to a reduced quadratic model (Equations (10) and (11)) and their statistical significance and validity were tested via the analysis of variance (ANOVA) detailed under Section 3.3.1. The model Equations (10) and (11) are expressed with the actual values of the input parameters (A, B, C), their interaction (AB, AC), and quadratic (B^2^, C^2^) terms as a function of the responses ($Y_{1}\;\mathrm{and}\;Y_{2}$). The positive sign represents the synergistic effect of the term on the response, whereas, the negative sign points out an antagonistic effect. In this view, the increasing order of the effect of the terms for the removal of colour follows AC > B^2^ > AB > B > A > C, whereas that of the turbidity removal is AC > AB > B > C^2^ > A > C. Also, the optimal values of the parameters are obtained by estimating the regression equation and the analysis of the surface response:

(9) $$\mathrm{Color}\;(Y_{1})=148.575-8.438\;5A-1.04B-8.668C-0.163\;1\mathrm{AB}+2.288\;\mathrm{AC}+0.0256{\;B}^{2}$$

(10) $$\mathrm{Turbidity}\;(Y_{2})=143.429-9.3017\;A-0.2023B-9.472C+0.0876\mathrm{AB}+2.667\;\mathrm{AC}-1.807C^{2}$$

#### 3.3.1. Analysis of Variance (ANOVA)

The model robustness in this study is evaluated by both the coefficient of determination *R*^2^ and adjusted-*R*^2^ with an estimated difference of less than 0.2 (Table 3). The fit statistics, as presented in Table 3, show that the predicted *R*^2^ of 0.886 and 0.893 for color and turbidity, respectively, were in reasonable agreement with the adjusted *R*^2^ of 0.966 and 0.975, where their difference is less than 0.2, suggesting good predictability of the models. Considering the acceptable threshold value of *R*^2^, which should be greater than or equal to 0.8, it becomes more relevant when it is closer to 1 [27,28]. The statistical significance (*p* < 0.05) of the model and its validity are tested by using the ANOVA (Table 4 and Table 5), with additional information such as the Fisher variation ratio (F-value), adequate precision, coefficient of variance (CV), probability value (Prob > F) and lack of fit. As shown in Table 4 and Table 5, the F-value and Prob > F values for the models and their independent parameters were found to be significant because of their *p*-values < 0.05. Also, there is only a 0.01% chance that an F-value this large could occur due to noise. Therefore, model terms with their *p*-values > 0.05 (insignificant), were not considered via model reduction to improve the models because they are not required to support the model hierarchy [34].

#### 3.3.2. Effects of the Factors and Their Interaction on the Responses

**Figure 4:**
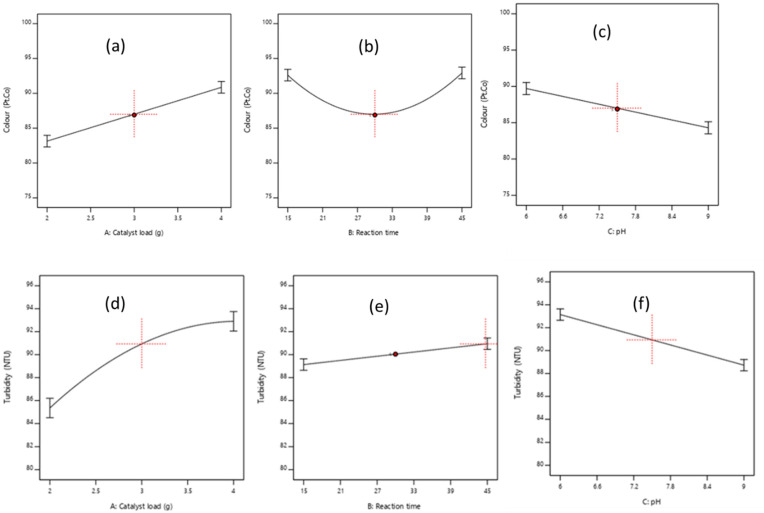
Response representation of individual factors effects on color (**a**–**c**) and turbidity (**d**–**f**) removal.

To study the interactive effects of the factors on the response, first one factor was varied at a time while keeping the other two process variables constant. Figure 4a–f shows the visual representation effects of each factor on the response. Figure 4a,d elucidate the effects of the catalyst dosage (2–4 mg/L) on the decrease of colour and turbidity, respectively. Decomposition efficiency increases with an increase in catalyst load up to 3.5 mg/L, beyond which there is a gradual reduction in terms of the removal of turbidity (Figure 4b). Thus, the more active sites made available in turn increases the rate of radical formation which increased the efficiency [35,36]. However, the overdosed catalyst load contributed to the turbidity of the solution by retarding the light penetration to activate the catalyst for collision with the colloids [35,36]. This resulted in the reduction of the turbidity efficiency (Figure 4b). Figure 4b,e, present the development of irradiation time which caused high organics degradation in terms of colour and turbidity, respectively. Mineralization of colour became less efficient within 20–30 min before increasing at 45 min, which might be due to the probability of slow reaction and deactivation of the active sites caused by the complex intermediaries with OH radicals [35,37]. Conversely, increasing the pH (6–9) decreased the colour (Figure 4c) and turbidity (Figure 4f) efficiency. This affirms that pH affects the pollutant charge neutralization with higher efficiency in the acidic medium (pH < 6) than the neutral (pH = 7) and alkaline conditions (pH > 7) [8,27].

**Figure 5:**
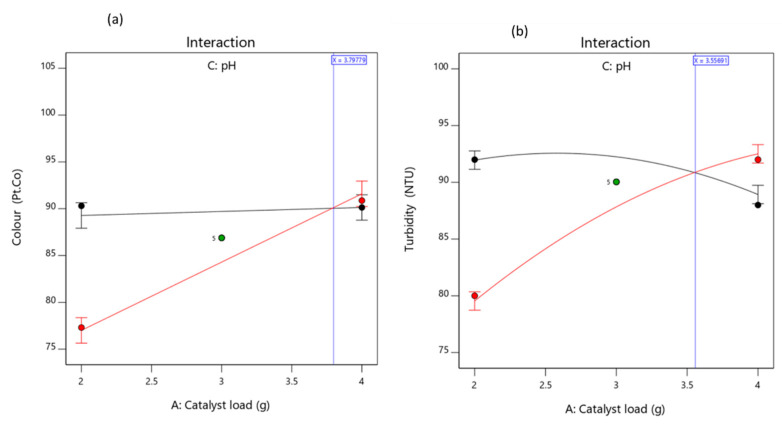
Interactive effect of catalyst load and pH on (**a**) color and (**b**) turbidity removal.

**Figure 6:**
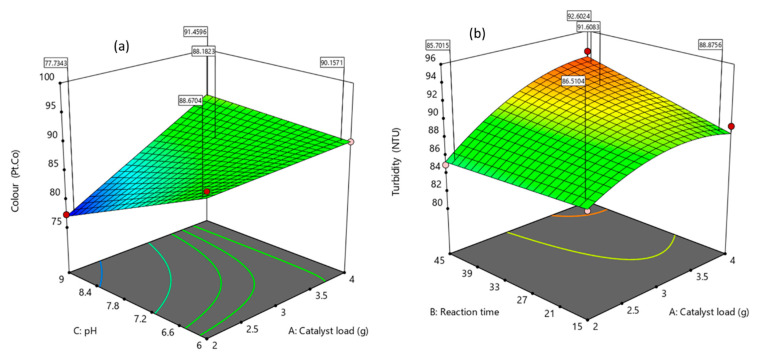
Response surface 3D plot of catalyst load and pH effects on (**a**) color and (**b**) turbidity removal.

Figure 5 and Figure 6 also confirmed that the interaction relationship between the catalyst load and pH has a significant impact on the photocatalytic activity on both responses (colour and turbidity). The three-dimensional response (3D) surface (Figure 6) was plotted based on the models (10) and (11) predicted removal efficiency, whereas, the reaction time was kept constant. This confirms that the interactive effects (AC) are predominant in comparison with the individual (A and C) and quadratic effects (A^2^, C^2^). This is in agreement with other reported studies, whereby the catalyst dosage can be considered as the most influential factor.

#### 3.3.3. Numerical Optimization

**Figure 7:**
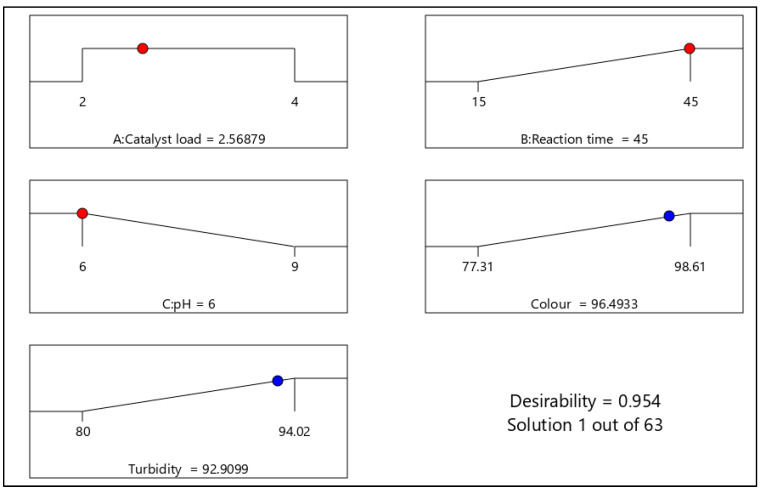
Ramp plot of numerical optimized conditions with the desirability of 95%.

To maximize the pollutant abatement, the Design-Expert software numerical optimization was carried out by setting individual factor values within their respective range such as catalyst load (2–4 g), reaction time (15–45 min) and pH (6–9), whereas the responses (colour and turbidity) were set at maximum with 95% confidence level. The desirability function approach was carried out, where the optimized conditions obtained are presented in Figure 7. In Table S1, out of 63 sets of solutions obtained, the most desirable (95.4%) and optimum condition selected is presented in Figure 7. Thus an optimum catalyst load of 2.6 g, a reaction time of 45 min and pH of 6 that resulted in 96.5% and 92.9% of colour and turbidity removal, respectively, was established. Additional optimum conditions with parametric constraints and desirability are presented in Table S1. Furthermore, the optimum conditions obtained were confirmed and verified experimentally which was closer to the predicted results with less than 2% deviation. This is agreeable to other studies suggesting RSM is economically viable for experimental optimizations based on its predictability with precision [27,28].

## 4. Conclusions

This study presented a co-precipitate of TiO_2_ impregnated on activated carbon (AC-TiO_2_) as a potential photocatalyst for wastewater treatment by optimizing the operating conditions (catalyst load, reaction, and pH) via response surface methodology (RSM). The crystal structure and functional groups were revealed by XRD and FTIR analysis. Furthermore, comparative performance and kinetic degradability between the AC-TiO_2_ and raw TiO_2_ were explored based on chemical oxygen demand (COD) reduction. First-order kinetic rate constants were estimated as 0.0322 min^−1^ and 0.0366 min^−1^ for TiO_2_ and AC-TiO_2_, respectively, with regression coefficient (*R*^2^) values of 0.9589 and 0.9828.

Results obtained by the Box-Behnken design (BBD) matrix were employed for modelling and optimization of the aforementioned operating conditions on photocatalytic degradation of colour and turbidity. A reduced quadratic model was developed as a function of the three input parameters and the responses were seen to be highly significant (*p* < 0.005). The models showed good predictability with a high correlation between the experimental values and the model-predicted values at a 95% confidence level. Catalyst load and pH was found to be the factors with the most influential impact on the response. The optimal conditions were found at a catalyst load of 2.6 g, reaction time of 45 min, and pH of 6, respectively, for 96.5% and 92.9% of colour reduction and turbidity removal via a numerical optimization. The optimal conditions were verified and confirmed experimentally to show less than 2% deviation. This confirms the use of BBD via RSM as viable for optimization and modelling photocatalytic systems in wastewater settings.

## Figures and Tables

**Table 1:** Box-Behnken design matrix for input variables.

Factor	Low Level (−1)	Medium (0)	High (+1)	Ref
A: Catalyst load (g)	2	3	4	[24]
B: Reaction time (min)	15	30	45	[26]
C: pH	6	7.5	9	[28]

**Table 2:** Results of Box-Behnken design with experimental and predicted values.

	Factor 1	Factor 2	Factor 3	Y_1_: Colour (%)		Y_2_: Turbidity (%)	
**Run**	**A: Catalyst Load (g)**	**B: Reaction Time (min)**	**C:pH**	**Exp**	**Pred**	**Exp**	**Pred**
1	2	15	7.5	85	86.32	86	86.16
2	3	30	7.5	86.88	87.00	90.04	90.04
3	4	30	9	90.87	91.59	92	92.51
4	2	30	9	77.31	77.00	80	79.54
5	3	45	9	88.98	90.22	88.08	88.74
6	3	30	7.5	86.88	87.00	90.04	90.04
7	2	30	6	90.3	89.28	92	91.96
8	3	30	7.5	86.88	87.00	90.04	90.04
9	4	30	6	90.13	90.13	88	88.93
10	4	15	7.5	98.61	98.90	89.31	88.50
11	2	45	7.5	91.92	91.50	85	85.35
12	3	30	7.5	86.88	87.00	90.04	90.04
13	3	45	6	95	95.63	94.02	93.16
14	4	45	7.5	95.8	94.35	93.57	92.95
15	3	30	7.5	86.88	87.00	90.04	90.04
16	3	15	6	95.09	95.31	90.9	91.34
17	3	15	9	91.73	89.90	87.18	86.92

**Table 3:** Analysis of variance (ANOVA) results for the response quadratic models.

Parameter	Colour	Turbidity
Standard deviation	1.06	0.63
Mean	89.71	89.19
Coefficient of variance (CV, %)	1.18	0.71
Coefficient of determination (*R*^2^)	0.972	0.988
Adjusted *R*^2^	0.966	0.975
Predicted *R*^2^	0.886	0.893
Adequate precision	32.33	33.67

**Table 4:** Analysis of variance (ANOVA) results for color quadratic models.

Source	Sum of Squares	Degree of Freedom	Mean Square	F-Value	*p*-Value	
Model	389.44	6	64.91	58.28	<0.0001	significant
A-Catalyst load	119.20	1	119.20	107.02	<0.0001	
B-Reaction time	0.2016	1	0.2016	0.1810	0.0495	
C-pH	58.48	1	58.48	52.51	<0.0001	
AB	23.67	1	23.67	21.25	0.0010	
AC	47.13	1	47.13	42.31	<0.0001	
B^2^	140.77	1	140.77	126.39	<0.0001	
Residual	11.14	10	1.11			
Lack of Fit	11.14	6	1.86	12.32	0.132	Non-significant
Pure Error	0.0000	4	0.0000			
Cor Total	400.58	16				

**Table 5:** Analysis of variance (ANOVA) results for the turbidity quadratic models.

Source	Sum of Squares	Degree of Freedom	Mean Square	F-Value	*p*-Value	
Model	179.76	6	29.96	75.42	<0.0001	significant
A-Catalyst load	49.40	1	49.40	124.36	<0.0001	
B-Reaction time	6.62	1	6.62	16.68	0.0022	
C-pH	38.98	1	38.98	98.14	<0.0001	
AB	6.92	1	6.92	17.41	0.0019	
AC	64.00	1	64.00	161.11	<0.0001	
A^2^	13.83	1	13.83	34.82	0.0002	
Residual	3.97	10	0.3972			
Lack of Fit	3.97	6	0.6621	10. 52	0.154	Non-significant
Pure Error	0.0000	4	0.0000			
Cor Total	183.73	16				

## Data Availability

Not applicable.

## Supplementary Materials

The following are available online, Table S1. Numerical optimized conditions.
