# Supplementary material for: Response Surface Methodology: Photocatalytic Degradation Kinetics of Basic Blue 41 Dye Using Activated Carbon with TiO2
Source: Molecules. 2021 Feb 18;26(4):1068. doi: 10.3390/molecules26041068 (PMC7922207; doi:10.3390/molecules26041068)
Supplement: Supplementary file 1 [file molecules-26-01068-s001.pdf]

molecules

Supplementary Materials

Response Surface Methodology: Photocatalytic Degradation Kinetics of Basic Blue 41 Dye Using Activated Carbon with TiO_2

Emmanuel Kweinor Tetteh *, Elorm Obotey Ezugbe, Dennis Asante-Sackey, Edward Kwaku Armah and Sudesh Rathilal

Green Engineering and Sustainability Research Group, Department of Chemical Engineering, Faculty of Engineering and The Built Environment, Durban University of Technology, Durban 4001, South Africa; elormezugbe.ee6@gmail.com (E.O.E.); ingsackey@gmail.com (D.A.-S.); edwardkarmah@gmail.com (E.K.A.); rathilals@dut.ac.za (S.R.)

* Correspondence: ektetteh34@gmail.com or emmanuelk@dut.ac.za

Highlights:

- The mechanism of incorporating activated-carbon as a co-photocatalyst to enhance wastewater treatment is discussed.
- Characteristics of TiO_2 doped activated carbon photocatalyst.
- Photocatalytic degradation of Basic Blue 41 dye organics (COD) is highlighted.
- Optimization via response surface methodology was elucidated.

Table S1. Numerical optimized conditions.

Number	Catalyst load	Reaction time	pH	Colour	Turbidity	Desirability	
**1**	**2.569**	**45.000**	**6.000**	**96.493**	**92.910**	**0.954**	**Selected**
2	2.567	45.000	6.000	96.496	92.908	0.954	
3	2.583	45.000	6.000	96.464	92.929	0.954	
4	2.560	45.000	6.000	96.511	92.898	0.954	
5	2.592	45.000	6.000	96.448	92.940	0.954	
6	2.547	45.000	6.000	96.537	92.879	0.954	
7	2.605	45.000	6.000	96.421	92.957	0.954	
8	2.620	45.000	6.000	96.391	92.974	0.954	
9	2.625	45.000	6.000	96.381	92.980	0.954	
10	2.640	45.000	6.000	96.351	92.997	0.954	
11	2.493	45.000	6.000	96.644	92.797	0.954	
12	2.658	45.000	6.000	96.315	93.017	0.954	
13	2.693	45.000	6.000	96.244	93.051	0.954	
14	2.715	45.000	6.000	96.199	93.070	0.954	
15	2.727	45.000	6.000	96.176	93.080	0.953	
16	2.414	45.000	6.000	96.804	92.656	0.953	
17	2.553	45.000	6.005	96.510	92.873	0.953	
18	2.380	45.000	6.000	96.871	92.590	0.953	
19	2.801	45.000	6.000	96.027	93.128	0.952	
20	2.812	45.000	6.000	96.005	93.134	0.952	
21	2.391	44.944	6.000	96.801	92.610	0.952	
22	2.320	45.000	6.000	96.991	92.461	0.952	
23	2.663	44.853	6.000	96.183	93.017	0.951	
24	2.262	45.000	6.000	97.108	92.323	0.951	
25	2.897	45.000	6.000	95.835	93.161	0.951	
26	2.945	45.000	6.000	95.739	93.165	0.949	
27	2.204	45.000	6.000	97.224	92.175	0.949	
28	2.654	44.740	6.000	96.106	93.005	0.949	

29	2.197	45.000	6.000	97.240	92.154	0.949
30	2.628	44.721	6.000	96.142	92.976	0.949
31	2.993	45.000	6.000	95.644	93.161	0.948
32	2.151	45.000	6.000	97.330	92.028	0.948
33	2.854	45.000	6.029	95.860	93.097	0.947
34	2.813	44.738	6.000	95.793	93.122	0.947
35	2.135	45.000	6.000	97.363	91.981	0.947
36	2.668	44.591	6.000	95.957	93.014	0.946
37	3.056	45.000	6.000	95.517	93.143	0.946
38	2.105	45.000	6.000	97.423	91.891	0.946
39	2.840	45.000	6.063	95.813	93.026	0.943
40	3.172	45.000	6.000	95.284	93.072	0.942
41	2.000	45.000	6.006	97.609	91.528	0.940
42	2.152	44.403	6.000	96.791	92.038	0.937
43	3.287	45.000	6.000	95.053	92.953	0.937
44	3.299	45.000	6.000	95.029	92.938	0.936
45	3.317	45.000	6.000	94.993	92.915	0.935
46	2.722	43.913	6.000	95.321	93.036	0.933
47	3.295	45.000	6.039	94.993	92.917	0.932
48	3.378	45.000	6.000	94.870	92.825	0.932
49	3.470	45.000	6.000	94.686	92.666	0.927
50	3.196	44.573	6.145	94.725	92.880	0.916
51	3.629	45.000	6.000	94.367	92.319	0.916
52	3.650	45.000	6.000	94.325	92.266	0.914
53	3.660	45.000	6.000	94.304	92.240	0.914
54	3.448	45.000	6.183	94.588	92.656	0.911
55	3.709	45.000	6.000	94.206	92.108	0.910
56	3.739	45.000	6.030	94.144	92.040	0.905
57	3.326	45.000	6.286	94.674	92.731	0.905
58	3.575	43.757	6.040	93.644	92.312	0.893
59	3.964	45.000	6.000	93.695	91.286	0.887
60	2.000	44.999	6.468	95.718	89.615	0.841
61	4.000	45.000	6.880	94.051	92.205	0.834
62	4.000	45.000	6.919	94.070	92.251	0.831
63	2.000	30.948	6.000	89.464	91.932	0.713
